# Oxidative low-density lipoprotein and shear induced calcification within a calcific aortic valve disease-on-a-chip platform

**DOI:** 10.3389/fcvm.2025.1655341

**Published:** 2025-10-23

**Authors:** Melissa Mendoza-Seale, Mei-Hsiu Chen, Peter Huang, Gretchen J. Mahler

**Affiliations:** ^1^ Department of Biomedical Engineering, Binghamton University, Binghamton, NY, United States; ^2^ Department of Mathematics and Statistics, Binghamton University, Binghamton, NY, United States; ^3^ Department of Mechanical Engineering, Binghamton University, Binghamton, NY, United States

**Keywords:** lipoprotein, shear stress, aortic valve, calcification, microfluidics, microphysiological system

## Abstract

**Background:**

Early-stage calcific aortic valve disease (CAVD) has been characterized by the infiltration of immune cells, reorganization of the extracellular matrix, and the deposition and oxidation of low-density lipoproteins (oxLDL). Worldwide studies have revealed that aortic valve disease accounts for up to 43% of patients exhibiting heart disease.

**Methods:**

We utilized a CAVD-on-a-chip platform of the aortic valve fibrosa to assess the hypothesis that culture calcification will increase with endothelial cell presence, increased oxLDL concentration (25 μg/ml or 50 μg/ml), and shear stress (20 dyne/cm_2_). CAVD chips consisted of collagen I hydrogels with porcine aortic valve interstitial cells embedded and porcine aortic valve endothelial cells seeded on top of the matrix for up to two days.

**Results:**

Here, we demonstrate that the presence of endothelial cells and shear stress drives alkaline phosphatase activity, sulfated glycosaminoglycan production, and the formation of mono-, di-, and octa- calcium phosphates, and hydroxyapatites. Two-day dynamic cultures showed 3D cell-oxLDL interactions, leading to extracellular matrix remodeling and endothelial dysfunction.

**Discussion:**

Given that CAVD has no targeted intervention, continued evolution of this CAVD-on-a-chip model sheds light on mechanisms in disease onset and can lead to significant contributions in preclinical drug development.

## 1 Introduction

Calcific aortic valve disease (CAVD) is the third most common heart disease in the Western world, following coronary heart disease and hypertension (1). CAVD is an active pathobiological process ranging from mild valve thickening (aortic sclerosis) to severe leaflet calcification (aortic stenosis) (2–4). Valve degradation begins with extracellular matrix (ECM) degradation when collagen and proteoglycans accumulate, and elastic fibers fragment and misalign. This fibrosis then causes the valve tissue to stiffen and eventually leads to restricted blood flow (5, 6). The stiffening of the leaflets is caused by the buildup of calcific nodules that form in the fibrosa layer and progress throughout (1). *In vitro*, CAVD has been described as a two-stage process: early-stage and late-stage. Early CAVD has been characterized by the infiltration of immune cells, reorganization of the ECM, and the deposition of oxidized low-density lipoproteins (oxLDL) (6).

Given that there is no targeted therapy for CAVD on the market, researchers are investigating disease mechanisms in both onset and progression to develop new drug interventions. Clinical factors associated with CAVD include age, male gender, serum lipoprotein(a) and low-density lipoprotein levels, height, hypertension, metabolic syndrome, and smoking (4). The deposition and oxidation of lipoproteins have been characteristically used to define early-stage CAVD disease progression (7). Specifically, early calcium deposition is found adjacent to lipoproteins in deep regions of the fibrosa (8). Valvular lipid studies have focused on several types of lipoproteins found in disease progression: lipoprotein(a) (9, 10), lipopolysaccharides (LPS) (11, 12), lysophosphatidylcholine (LPC) (13, 14), and oxLDL (11, 12, 15). Examination of diseased pig valves *in vivo* suggests that glycosaminoglycans (GAG) enrichment and oxLDL deposition occur prior to immune cell infiltration, giving rise to valve pathological events (6). Further, Nadlonek et al. identified that oxLDLs stimulate toll-like receptors (TLRs)-2 and -4 and promote aortic valve calcification in human aortic valve interstitial cells (VICs) *in vitro* (11). Zeng et al. treated human VICs with LPS, oxLDL, or LPS and oxLDL for 48 h, and found that the LPS-oxLDL combined treatment increased bone morphogenetic protein-2 (BMP-2) and alkaline phosphatase (ALP) activity; thereby, demonstrating that lipoproteins augment the osteogenic responses through modulation of Notch1 and NFκB activation (12). Yu et al. also identified that a 48 h exposure of 50 μg/ml lipoprotein(a) to human aortic VICs resulted in increased cell proliferation and increased ALP activity, while three-week exposure significantly increased calcium deposition (9). Yamashita et al. reported that oxLDLs further increased calcium phosphate sediments in the formation of ectopic calcification (15). Bouchareb et al. studied the effect of autotaxin, which is transported by lipoprotein(a), and LPC on mineralization by stimulating the NFκB/Interleukin-6(IL-6)/BMP pathway in VICs (13). Further, comparison of proteomic analysis of lipoprotein(a) proteome from aortic stenosis patients and transcriptomic analysis of explanted calcified valves identified the most enriched pathways involved cellular aging, chondrocyte development, and inflammation (10). Wiltz et al. examined the effect of LPC, a bioactive lysophospholipid commonly found in low-density lipoproteins, on valvular cell mineralization, highlighting that aortic valvular cultures treated with LPC had increased phosphate mineralization, ALP activity, calcium content, and apoptosis (14).

Additionally, CAVD pathogenesis can induce abnormal hemodynamic forces and ECM remodeling, alter cell proliferation and morphology, and initiate apoptosis (1, 16–21). Early disease progression markers focus on ECM remodeling, specifically increased cell proliferation, and detection and accumulation of proteins (ex. BMP-2, ALP, and GAGs) (9, 12, 22–27). ALP activity promotes calcification and mineralization by reducing pyrophosphate and osteopontin via hydrolysis (20, 21, 28). For example, Rajamannan et al. demonstrated that an increase in ALP protein contributed to CAVD in human calcified tissues (25). Detection of proteoglycans rich in acidic, sulfated, and extracellular GAGs has been reported in cardiovascular calcifications, using histochemical reagents such as Alcian Blue (AB), Cuprolinic blue, and/or Cupromeronic blue (23). Porras et al. demonstrated that lipoprotein deposition increased VIC deposition of GAGs quantified via an AB assay, leading to inflammatory activity and disease progression (6). Further, Dahal et al. and Bramsen et al. showed an increase in GAG and collagen-I production by mesenchymally-transformed endothelial cells (24, 26).

Here, we utilized a 3D microfluidic platform of the aortic valve fibrosa layer to study the early onset of CAVD in two-day cultures. We assessed the hypothesis that culture calcification will increase with endothelial cell presence, increased oxLDL concentration, and shear stress. This hypothesis was tested by comparing static (no shear) and dynamic (20 dyne/cm^2^ shear stress) 3D hydrogel cultures via (1) quantitative analysis of ALP activity and GAG production normalized to protein content, (2) scanning electron microscopy with energy dispersive x-ray spectroscopy analysis (SEM/EDX), and (3) fluorescent microscopy.

## 2 Materials and methods

Fabricated as previously described in Mendoza et al., the CAVD-on-a-chip devices utilized soft lithography and plasma bonding, and contained an internal 3D hydrogel matrix (19). Disease progression was assessed using a colorimetric ALP assay, detection of sulfated GAG production via an AB assay, and SEM/EDX. oxLDL-cell interactions were examined with confocal microscopy. The following sections provide detailed materials and methods.

### 2.1 Device design and fabrication

**Figure 1:**
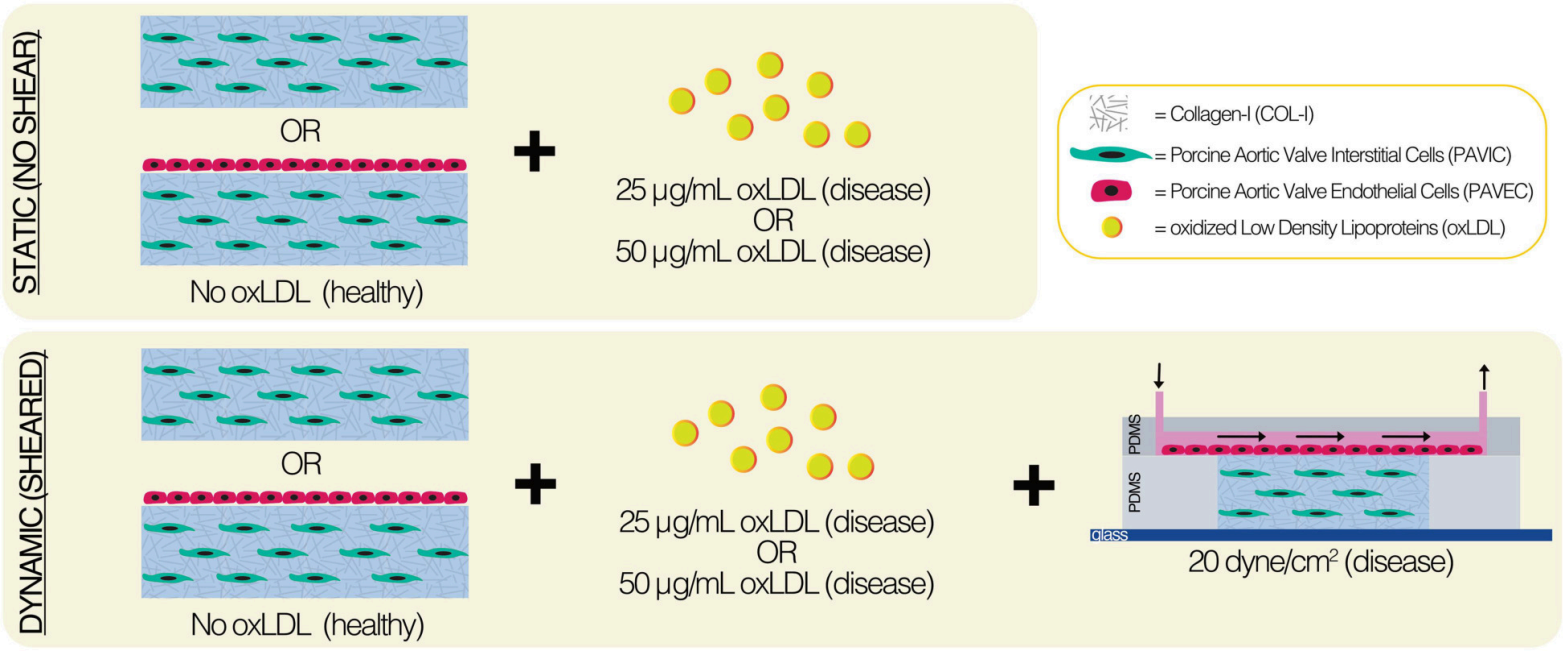
Experimental methods of static and dynamic aortic valve fibrosa model.

Fabrication methods (19) and device design (29) have been previously described and characterized. Briefly, the flow channel was created using a fabricated silicon wafer mold by photolithography with HARESQ-50 (KemLab) negative photoresist. After which, 9:1 polydimethylsiloxane (PDMS) (Sylgard-184, Dow Corning) was used to cast against the silicon wafer mold to create the flow channel and hydrogel chamber layers of the device. A corona discharge device was then used to permanently bond the PDMS layers with a flat glass substrate (Figure 1). Internal device surfaces were prepared using 50 μg/ml poly-D-lysine (PDL) and 50 μg/ml Cell-TAK™ treatments prior to the introduction of biologics.

### 2.2 Primary cell culture

Models utilized porcine aortic valve interstitial cells (PAVIC) and/or porcine aortic valve endothelial cells (PAVEC) isolated from tissues obtained at local abattoirs, as previously described in the literature (30). PAVIC (passage 3–4) were grown in Dulbecco's Modified Eagle medium (DMEM) (Gibco Life Tech) supplemented with 10% fetal bovine serum (FBS) (VWR) and 1% penicillin-streptomycin (Gibco Life Tech). PAVEC (passages 4–5) were grown in a 50 μg/ml collagen-I (COL-I)-coated flasks (Corning) in DMEM supplemented with 10% FBS, 1% penicillin-streptomycin, and 50 U/ml heparin sulfate (Sigma-Aldrich) prior to seeding experiments.

### 2.3 oxLDL integration

Fluorescent oxLDLs (Dil-oxLDL, ThermoFisher) were used to visualize cell-oxLDL interactions, and non-fluorescent oxLDLs (ThermoFisher) were used for non-imaging purposes at several concentrations. For static and dynamic experiments, COL-I was treated with 25 μg/ml, 50 μg/ml and 200 μg/ml Dil-oxLDL or non-fluorescent oxLDL (ThermoFisher) for two days prior to hydrogel fabrication and experimental set-up. Following oxLDL treatment of COL-I, hydrogels were fabricated with valve cells and incubated at 37 °C and 5% CO_2_ for up to 2 days. Hydrogels were washed 3 times with 1X phosphate-buffered saline (PBS), fixed with 4% paraformaldehyde (PFA) overnight, and washed again 3 times with 1XPBS. Samples were then permeabilized using 0.2% TritonX-100 (Sigma Aldrich) solution for 10 min, washed 3 times with 1XPBS, and stained with CellMask™ Deep Red Plasma membrane stain (1:1,000 dilution) (ThermoFisher) and Hoechst 33432 DNA stain (5 μg/ml dilution) (ThermoFisher) for 1 h with gentle agitation. The staining mixture was removed and the gels were rinsed once with non-sterile 18 MΩ water prior to imaging. Hydrogels were imaged utilizing confocal laser scanning microscopy with a 40X water immersion lens (LSM 880, Zeiss).

### 2.4 Hydrogel fabrication

The 3D ECM was made by mixing a PAVIC pellet at 1 × 10^6^ cells/ml with sterile 3XDMEM, 18 MΩ water, FBS, 0.1 M sodium hydroxide (Sigma-Aldrich), and rat-tail COL-I (Corning) on ice, in corresponding order. Hydrogels consisted of 1.5 mg/ml COL-I-only healthy controls or 1.5 mg/ml COL-I with either 25 μg/ml or 50 μg/ml oxLDL concentration (ThermoFisher). Both static and dynamic conditions were used and were placed at 37 °C and 5% CO_2_ for up to two days. In static conditions, PAVIC-embedded 300 μl hydrogels were seeded into 50 μg/ml Cell-TAK™ pre-treated (Corning) 24-well plates (Corning). After one hour, PAVEC were seeded onto the matrix at 95,000 cells/cm^2^ in 400 μl PAVIC medium. In dynamic conditions, the PAVIC-embedded (1 × 10^6^ cells/ml) hydrogel was injected into the pre-treated PDMS middle layer utilizing a 23G sterile needle (BD) and allowed to crosslink for one hour. PAVEC (95,000 cells/cm^2^) were introduced onto the matrix via microchannel inlet and allowed to attach for 4 h prior to the flow initiation. Each device was then connected to a peristaltic pump (205S, Watson Marlow) using 0.51 mm inner diameter tubing (Cole-Palmer) and 0.79 mm inner diameter connector tubing (Cole-Palmer). Steady shear stress of 20 dynes/cm^2^ was applied to the top of the matrix by controlling the flow rate. A recirculating 500 μl reservoir of PAVIC medium was used to maintain dynamic cultures.

### 2.5 ALP activity assay

Early disease progression markers were assessed using ALP activity assay after two-day static and dynamic cultures. Hydrogels were washed three times with 1XPBS and individually digested in 400 μl of collagenase solution at 37 °C and 5% CO_2_. Collagenase solution was prepared sterilely using collagenase type II (Worthington Biochem) at 600 units/ml in DMEM (Gibco). Once fully digested, samples were microcentrifuged at 106 × g for 5 min, the supernatant was removed, and the pellet was resuspended in 1XPBS and microcentrifuged at 106 × g for 5 min again. Subsequently, 1XPBS supernatant was removed and samples were resuspended in 100 μl of sterile 18 MΩ water. Samples were subjected to bath sonication at 4 °C for 15 min, during which a p-Nitrophenyl Phosphate (pNPP) solution was made utilizing one pNPP tablet (Sigma-Aldrich) and one Tris buffer tablet (Sigma-Aldrich) dissolved in 5 ml of 18 MΩ water. A standard calibration curve was made using a serial dilution of 10 mg/ml p-nitrophenol (4-nitrophenol, Sigma-Aldrich) solution in 18 MΩ water into pNPP solution. Using a 96-well plate, 85 μl of pNPP solution was added to each well, and 25 μl of each standard solution in triplicate or sonicated sample lysate was added to each well. The microwell plate was incubated at room temperature for one hour, after which colorimetric detection of p-nitrophenol was assessed using a plate reader at 405 nm in Gen5™ (Synergy 2, BioTek). The standard curve was further utilized to detect mg/ml of p-nitrophenol in samples and normalized to the Bradford assay to obtain mg of p-nitrophenol per mg of cell protein.

### 2.6 Bradford assay

A Bradford assay was performed to determine the concentration of cell protein in each sample, and to normalize ALP activity and AB results. Negative controls were assessed, where static COL-I control hydrogels were 300 μl in a 24-well plate and dynamic COL-I control hydrogels were 100 μl in a 96-well plate without cells and with varying concentrations of oxLDLs (0 μg/ml, 25 μg/ml, 50 μg/ml). Remaining sample lysate from the ALP procedure was sonicated for an additional 15 min at 4 °C for the detection of protein. A standard calibration curve was made using a serial dilution of 1 mg/ml bovine serum albumin (VWR) in 18 MΩ water into Bradford reagent (Sigma-Aldrich). Using a 96-well plate, 250 μl of Bradford reagent was added to each well, and 5 μl of each standard solution in triplicate or sonicated sample lysate (30 min total) was added to each well. The microwell plate was incubated at room temperature for 15 min, after which colorimetric detection of protein was assessed using a plate reader at 595 nm. The standard curve was then utilized to detect mg/ml of cell protein in samples.

### 2.7 AB quantification assay

Early disease progression markers were assessed using an AB assay for the detection of sulfated glycosaminoglycan production in culture medium (6, 31). Following the two-day cultures, static culture medium and dynamic culture medium were collected from the well plate or the recirculating reservoir, respectively. A 10 mg/ml AB (8GX, Alfa Aesar) solution was prepared using a 1/100 dilution of AB into 3% acetic acid (Amresco). The pH was adjusted to 2.5, and a fresh working solution was made for every assay: 10% of the stock solution with 0.25% of TritonX-100 (Sigma Aldrich). Medium samples were stained with 100 μl of working solution and microcentrifuged for 10 min at 20,800 × g and 4 °C. After aspirating the supernatant, the pellet was dissolved in 500 μl of 5 M hydrochloric acid for 10 min at room temperature. The pellets were then subjected to resuspension using pipetting and vortex, and again microcentrifuged for three minutes at 20,800 × g and 4 °C. A standard calibration curve was made using a serial dilution of 10 mg/ml AB in 3% acetic acid. Using a 96-well plate, 150 μl of each standard in triplicate and 150 μl of sample supernatant was added to each well. Colorimetric detection of AB was assessed using a plate reader at 600 nm. The standard curve was then utilized to detect mg/ml of AB in samples and normalized to the Bradford assay to obtain mg of AB per mg of protein.

### 2.8 SEM/EDX

As previously described in Mendoza et al. (19), disease progression was also assessed with SEM/EDX, an imaging and spectroscopy technique used to analyze microstructure and elemental composition (19, 32–35). Following two days of experimentation, static and dynamic hydrogels were washed three times with 1XPBS, fixed with 4% PFA overnight, and washed again 3 times with 1XPBS. Samples were subjected to ethanol (Koptec) dehydration for 20 min from 50% ethanol in 18 MΩ water to 100% ethanol. Samples were then subjected to Hexamethyldisilazane (HMDS) (Sigma-Aldrich) dehydration for 20 min in each concentration (1:2 HMDS:100% ethanol, 2:1 HMDS:100% ethanol, 100% HMDS) prior to final 100% HMDS immersion and were left overnight until the sample dried out in the fume hood. Samples were mounted onto aluminum sample holders with carbon tape (Electron Microscopy Sciences) and prepared with at least 15 nm of fresh carbon sputter (Cressington 208C, Ted Pella). Samples were imaged under SEM (FE-SEM Supra-55 VP, Zeiss) with the following parameters: 3–5 kV, 6–8 mm working distance, and the In-Lens detector. Images were obtained of calcified nodules, as well as of endothelial cells, to study the effect of oxLDLs on endothelial dysfunction of the cell membrane (2, 36–39). Images of individual cells (*n* ≥ 3) were used to further analyze membrane pore frequency, percent area of pores compared to the entire cell area, and individual pore areas with ImageJ Particle Analysis tool (40). EDX (Octane Elect Super C5) analyses were performed with the following SEM and software (EDAX APEX Advanced, Ametek) parameters: 15 kV, 15 mm working distance, and 10%–40% dead time. Data represented as *n* ≥ 17 measurements per condition in atomic percent (At%) and calculations of At% Ca/P were used to quantify calcium phosphate mineralization.

### 2.9 Fluorescent staining and imaging

Following two-day experimentation with 25 μg/ml or 50 μg/ml Dil-oxLDL, hydrogels were washed three times with 1XPBS, fixed with 4% paraformaldehyde (PFA) overnight, and washed again three times with 1XPBS. Samples were then permeabilized using 0.2% TritonX-100 (Sigma Aldrich) solution for 10 min, washed three times with 1XPBS, and all cells (PAVIC-only and PAVIC + PAVEC) were stained with CellMask™ Deep Red Plasma membrane stain (1:1,000 dilution) (ThermoFisher) and Hoechst 33432 DNA stain (5 μg/ml dilution) (ThermoFisher) for one hour with gentle agitation. Staining mixture was removed and gels were rinsed once with 18 MΩ water prior to imaging. Hydrogels were imaged utilizing confocal laser scanning microscopy with a 40X water immersion lens (LSM 880, Zeiss). Z-stacks were used to demonstrate orthogonal cross-sections of oxLDL uptake by valvular cells.

### 2.10 Statistical analysis

All data was presented as mean ± standard error of the mean (SEM), unless otherwise specified. Due to the small sample sizes and the use of ratios, Mann–Whitney tests were used to compare the rankings of ALP activity or GAG production relative to protein content control between static and dynamic conditions in varying experimental conditions. Kruskal–Wallis tests with Dunn's Multiple Comparisons *post-hoc* tests were used to compare Ca/P ratios and the characteristics of endothelial pores across multiple experimental conditions. A *p < 0.05 was considered statistically significant. Sample size for each experimental condition was specified in the methods and figure legends. Analyses were conducted in GraphPad Prism 8 (GraphPad).

## 3 Results

### 3.1 Shear stress drives ALP activity and sulfated GAG production

**Figure 2:**
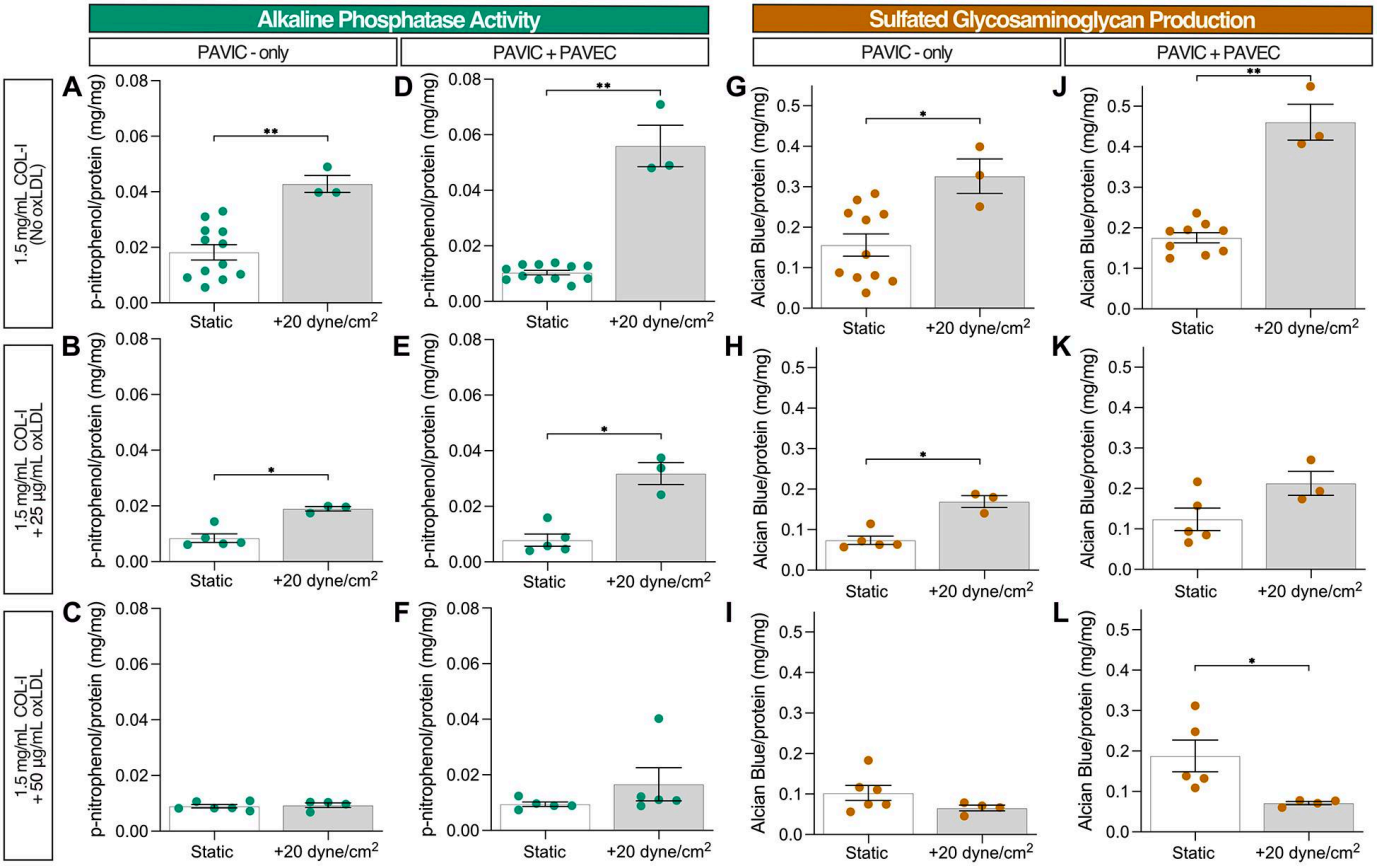
Early disease progression markers after 2 days in culture: static and dynamic collagen hydrogels with PAVIC and PAVIC/PAVEC co- culture. **(A–F)**. Alkaline phosphatase activity normalized to protein content via Bradford assay **(G–L)**. Sulfated glycosaminoglycan production and release into culture medium detected with Alcian Blue and normalized to protein content via Bradford assay. Data shown as mean ± SEM, where static (*n* ≥ 5 samples) and dynamic (*n* = 3 samples), and statistical significance shown according to two-sided Mann–Whitney test, *p < 0.05. (PAVIC, porcine aortic valve interstitial cells, PAVEC, porcine aortic valve endothelial cells, oxLDL, oxidative low-density lipoproteins).

Early disease progression markers, such as ALP activity detection and sulfated GAGs released into the media via AB detection, were assessed after two days in static and dynamic (20 dyne/cm^2^) samples. Increased ALP activity was detected at 20 dyne/cm^2^ compared to static controls, specifically in the presence of endothelial cells. However, the increase of oxLDL concentration inhibited ALP activity relative to protein content (Figures 2A–F). Similarly, sulfated GAG production relative to protein content detected in the medium increased in the presence of shear and endothelial cells but decreased as oxLDL concentration increased, except in the presence of 50 µg/ml oxLDL. The reduction in relative GAG production as oxLDL concentration increased was most pronounced in dynamic conditions (Figures 2G–L). Both ALP activity and GAG production were normalized to protein content controls containing the corresponding lipoprotein content, collagen hydrogel volume, and dynamic conditions; Supplementary Figure S1 demonstrates protein quantification via the Bradford assay, confirming increased protein concentration with increasing lipoprotein concentration in dynamic cultures.

### 3.2 SEM/EDX reveals hydroxyapatite formation in two-day dynamic cultures

**Figure 3:**
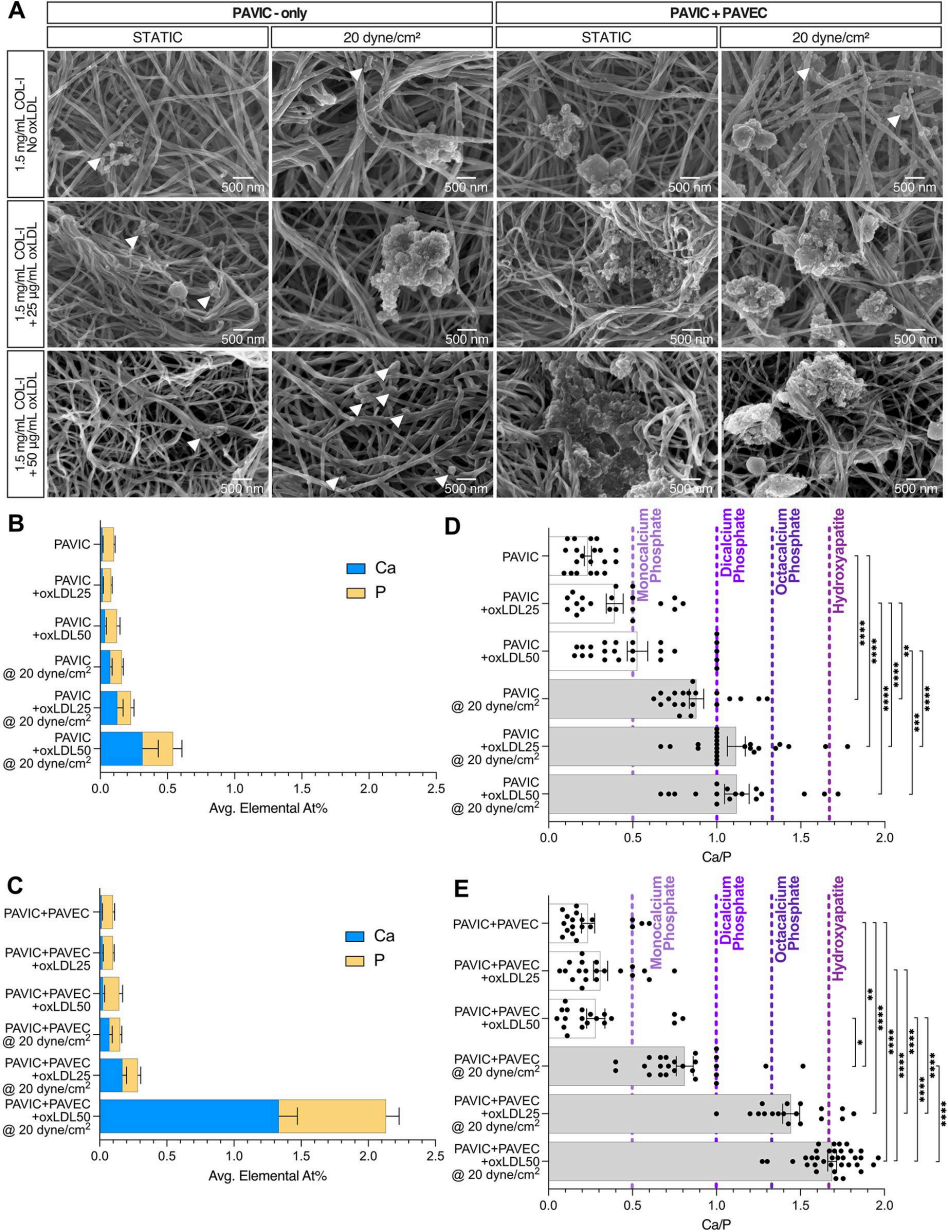
Hydroxyapatite formation qualification and quantification in both static and microfluidic cultures after 2 days, PAVIC-only and when co-cultured with PAVEC. **(A)**. Scanning electron microscopy (SEM) qualitative images with arrows indicating the presence of smaller nodules (scale = 500 nm). Energy dispersive x-ray spectroscopy (EDX) calcium and phosphorus elemental atomic percentages (At%) for **(B)**. PAVIC-only and **(C)**. PAVIC + PAVIC co-cultures, where Mean ± SEM, *n* ≥ 18 measurements per sample, and statistical significance shown in Supplementary Figure S3. Calcium phosphate mineralization based on EDX At% **(D)**. PAVIC-only and **(E)**. PAVIC + PAVEC co- cultures, where monocalcium phosphate calcium to phosphate ratio (Ca/P) = 0.5, dicalcium phosphate Ca/P = 1.0, octacalcium phosphate Ca/P = 1.33, and hydroxyapatite Ca/P = 1.67, Mean ± SEM, *n* ≥ 18 calculations per sample, and statistical significance shown according to Kruskal–Wallis with Dunn's multiple comparisons *post-hoc* test, *p < 0.05. (PAVIC, Porcine aortic valve interstitial cells; PAVEC, Porcine aortic valve endothelial cells; oxLDL25, 25 µg/ml oxidative low-density lipoproteins; oxLDL50, 50 µg/ml oxidative low-density lipoproteins; Ca/P, calcium to phosphorous ratio).

SEM/EDX was utilized to characterize culture calcification via calcium and phosphorous At%. SEM demonstrated that nodule microstructures were found embedded within the fibrous collagen matrix and localized around fiber bundles (Figure 3A). Qualitatively, increased calcified nodule formation was found in PAVIC + PAVEC cultures compared to PAVIC-only cultures, and with increasing oxLDL concentration. Supplementary Figure S2 demonstrates EDX analysis of dynamic conditions with and without endothelial cells and increasing oxLDL concentration. Spectra revealed the presence of several elements: carbon, silicon, aluminum, nitrogen, oxygen, calcium, phosphorous, and sulfur. Measurements indicated that the combination of 50 µg/ml oxLDL and shear in co-culture models resulted in significantly higher calcium and phosphorous content than in any other experimental condition (Figures 3B,C). Supplementary Figure S3 demonstrates elemental percentages of calcium and phosphorous for each condition, highlighting the compounding effect of oxLDL integration and the presence of shear stress. Ca/P ratios of EDX At% were calculated and plotted against a variety of well-studied pathological calcium phosphates leading to hydroxyapatite (Ca/P = 1.67) formation (Figures 3D,E). As previously described, Ca/P ratios were composed of a variety of calcium phosphates. In PAVIC-only cultures, static Ca/P ratios aligned with monocalcium phosphates (Ca/P = 0.5) and steadily increasing mineralization with increased oxLDL concentration. Dynamic Ca/P ratios aligned with dicalcium phosphates (Ca/P = 1.0) and octacalcium phosphates (Ca/P = 1.33): Ca/P = 0.879 ± 0.043 for control, Ca/P = 1.116 ± 0.054 at 25 µg/ml oxLDL, and Ca/P = 1.119 ± 0.073 at 50 µg/ml oxLDL (Figure 3D). In PAVIC + PAVEC cultures, static Ca/P ratios similarly aligned with monocalcium phosphates. Dynamic Ca/P ratios aligned with dicalcium phosphates, octacalcium phosphates, and hydroxyapatite increasing Ca/P with increasing oxLDL concentration: Ca/P = 0.811 ± 0.051 for control, Ca/P = 1.445 ± 0.050 at 25 µg/ml oxLDL, and Ca/P = 1.689 ± 0.026 at 50 µg/ml oxLDL (Figure 3E).

### 3.3 Fluorescent imaging demonstrates porcine cell-oxLDL interactions

**Figure 4:**
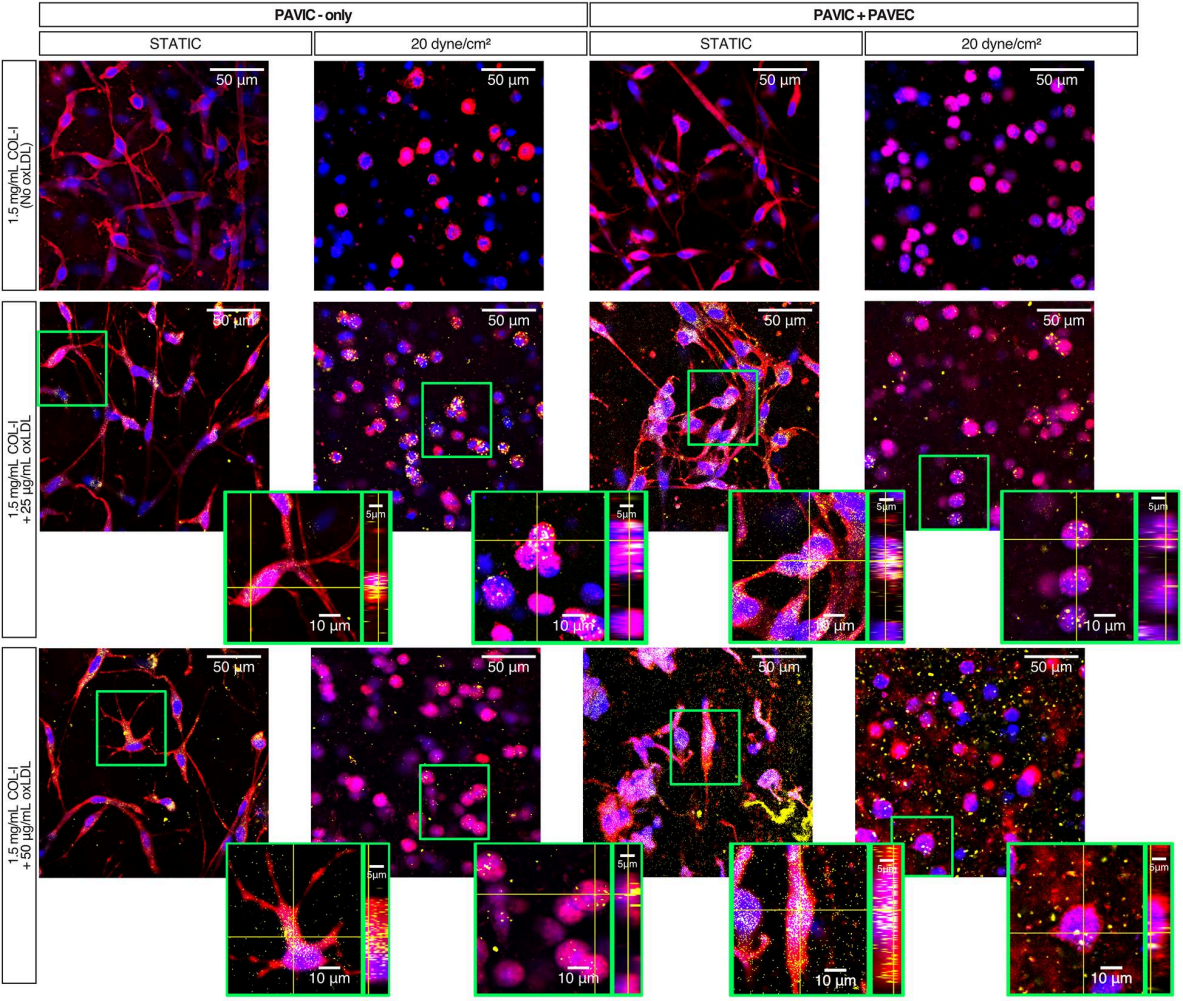
Static and dynamic oxLDL-cell interactions after 2 days in culture with PAVIC and PAVIC + PAVEC co-culture. Key: Plasma membrane (CellMask = Red), DNA (Hoechst = Blue), oxLDL (Dil-oxLDL = Yellow). (scale = 50 µm, highlighted box then zoomed into show individual cells orthogonally). (COL-I, Collagen-I; PAVIC, porcine aortic valve interstitial cells; PAVEC, porcine aortic valve endothelial cells; oxLDL, oxidative low-density lipoproteins).

Dil-conjugated oxLDLS were used to visualize oxLDL interactions with PAVIC-only and PAVIC + PAVEC co-cultures. Supplementary Figure S4 demonstrates that preliminary confocal imaging of co-cultured valve cells with a 200 µg/ml oxLDL concentration oversaturated porcine cell cultures, leaving 25 µg/ml and 50 µg/ml as experimental conditions. Further, Supplementary Figure S4B shows preliminary findings indicating that porcine cells remained viable in the high experimental conditions (50 µg/ml) in both static and dynamic co-cultures as shown by no cytotoxic effects in a Live/Dead assay. Qualitatively, fluorescent imaging demonstrated that when oxLDLs are cultured either with PAVIC alone or in PAVIC + PAVEC co-cultures, cells within the 3D matrix uptake these oxLDLs over two days in both static and dynamic systems (Figure. 4).

### 3.4 oxLDL treatment leads to endothelial dysfunction

**Figure 5:**
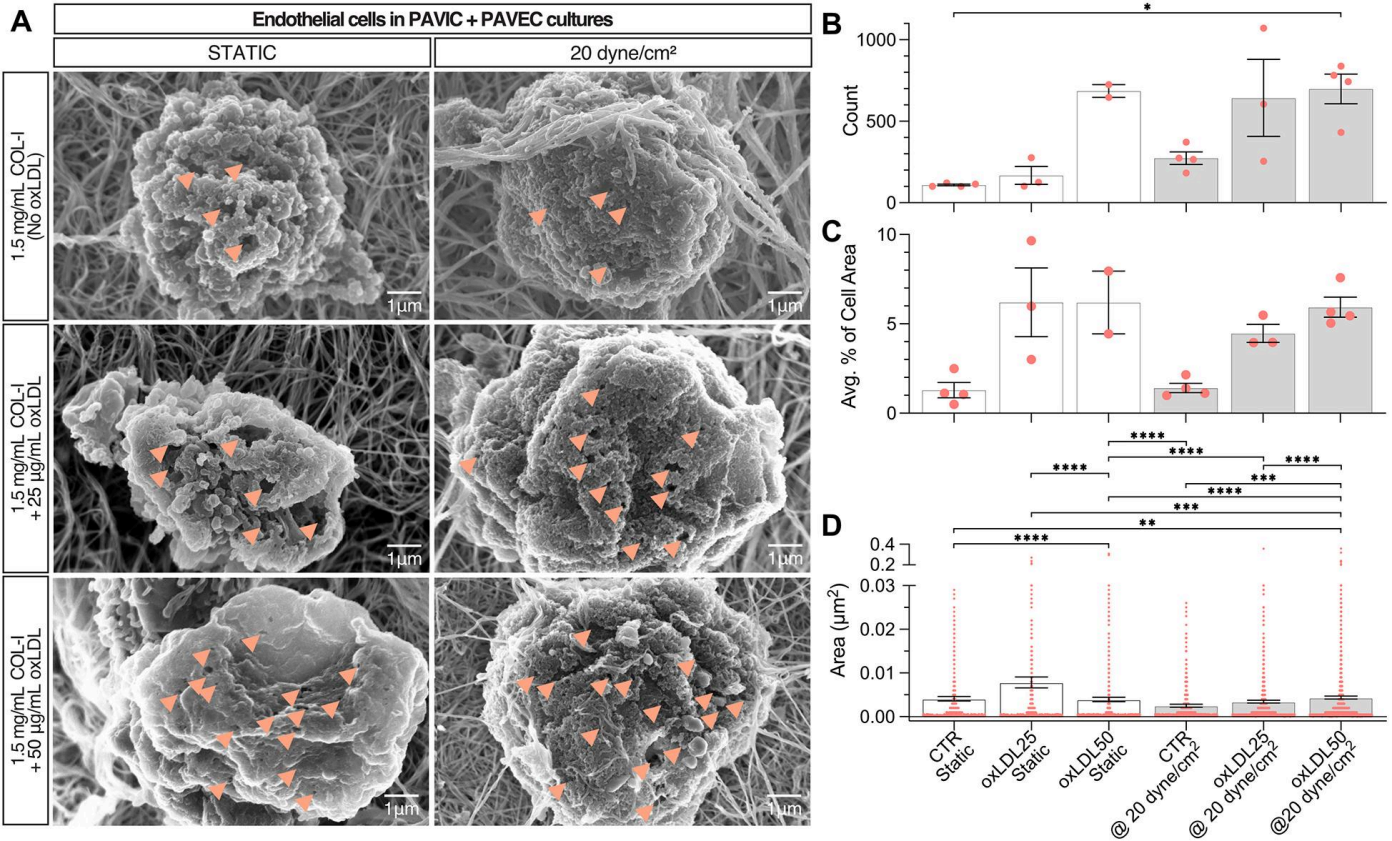
Endothelial dysfunction in static and dynamic PAVIC + PAVEC co-culture with oxLDL after 2 days. **(A)**. Scanning electron microscopy (SEM) qualitative images with arrows indicating the presence of smaller nodules (scale = 1 µm). Quantification of endothelial dysfunction as shown by **(B)**. Frequency of endothelial pores, **(C)**. Average percent area of pores compared to total cell area, and **(C)**. Area (µm^2^) of individual pores measured (*n* ≥ 400 measurements), where Mean ± SEM, *n* ≥ 3 images of cells analyzed, and statistical significance shown according to Kruskal–Wallis with Dunn's multiple comparisons *post-hoc* test, *p < 0.05. (PAVIC, Porcine aortic valve interstitial cells; PAVEC, Porcine aortic valve endothelial cells; oxLDL25, 25 µg/ml oxidative low-density lipoproteins; oxLDL50, 50 µg/ml oxidative low-density lipoproteins).

SEM images were obtained of endothelial cells from PAVIC + PAVEC co-cultures in both static and 20 dyne/cm^2^. Qualitatively, images indicated an increase in the presence of pores, or small holes in the plasma membrane, as a result of increasing oxLDL concentration (Figure 5A**)**. Quantitatively, ImageJ was used to count the frequency of pores, the total average percent area of pores compared to the total area of each cell, and the area of each individual pore. In both static and dynamic conditions, endothelial pores were found to be more frequent, increased in size, and take up higher % of cell area, as oxLDL concentration increased. Overall, in the presence of shear and increased oxLDL concentration, pores were more frequent and larger in size (Figures 5B–D).

## 4 Discussion

This study builds upon foundational work previously published in Mendoza et al. (19), involving the implementation of the CAVD-on-a-chip model to study cellular interactions with lipoprotein deposition leading to potential disease progression. Here, we demonstrated that 50 µg/ml oxLDL treatment, 20 dyne/cm^2^ shear, and endothelial cell presence over two days resulted in hydroxyapatite formation within the model.

Lipoprotein deposition and oxidation are characteristic early predictors for CAVD onset (6). Several lipoproteins studied in valvular disease progression are lipoprotein(a), LPS, LPC, and oxLDL (9–15). As described in this study, we sought to identify the effect of oxLDL deposition on the development of calcification seen *in vitro* in early CAVD. Porras et al. (*in vitro* and *in vivo mice*), Nadlonek et al. (*in vitro* human), Yamashita et al. (*ex vivo* bovine), Cote et al. (*in vivo* human), and Syvaranta et al. (*ex vivo* human) utilized human-derived or recirculating oxLDL to understand the development valvular pathological events (6, 11, 15, 41, 42). Interestingly, Zeng et al. demonstrated that a combination treatment of oxLDL with LPS increased BMP, Notch1, and NFκB activation, and increased ALP activity in human VICs (12). LPC is formed during lipoprotein oxidation and constitutes up to 40% of the lipid content found in oxLDL (43). Bouchareb et al. (13), Wiltz et al. (14), and Wilson et al. (44) revealed that this bioactive molecule is capable of driving mineralization through increased deposition of calcium, increased ALP activity, and increased apoptosis in VIC cultures (16, 17, 45). Yu et al. studied the effects of lipoprotein(a) deposition on human VICs, showing similar increased ALP activity *in vitro* (9). In this model, we saw that ALP activity normalized to protein content decreased with increasing concentrations of oxLDL content in both static and dynamic cultures. We believe this is because the upregulation of osteogenic factors, such as ALP, is linked to the onset and progression of CAVD (9, 12, 14, 25, 45, 46) rather than mineralization, which was evident in our model. Mathieu et al. supplemented cells from isolated *ex vivo* human calcified tissue with organic phosphates and induced calcification (46); this study further identified a strong correlation between mineralization and ALP activity. Alternative ALP detection methods can also be explored, given that the enzymatic activity assay described here resulted in minimal detection: immuno-gold labelling followed by SEM imaging (25), a Alkphase-B assay kit for electrophoresis (45), colorimetric assay kit for detection of ALP in the medium (9), cytochemical staining (12), or a fluorometric assay kit such as SensoLyte® (14).

ECM production was also probed as an early disease progression marker. Yu et al. demonstrated that 48 h lipoprotein(a) treatment of human aortic VICs increased cell proliferation, intracellular and extracellular ALP activity, and ECM production (9). Specifically, GAG enrichment is an early hallmark for aortic valve disease (6, 22, 47). Porras et al. found the presence of significant leaflet thickening caused extensive ECM remodeling (ex. collagen disorganization, proteoglycan enrichment, and elastin fragmentation), lipid oxidation, and macrophage infiltration in *ex vivo* swine aortic valve tissue with familial hypercholesterolemia (22). Furthermore, another study by Porras et al. identified a positive feedback loop driving further GAG enrichment both in an inflammatory response and in VIC activation (6). Similar to that of ALP activity in this model, sulfated GAG production normalized to protein content decreased with increasing oxLDL content in both static and dynamic cultures, indicating a link between GAG deposition and early formation of mineralization.

Preliminary static work required the understanding of lipoprotein integration into the *in vitro* COL-I hydrogel platform: concentration-dependence (25, 50, or 200 µg/ml oxLDL), pre-treatment (48 h treatment of collagen prior to fabrication of hydrogels) vs. same day integration (introduction during hydrogel fabrication), spatial heterogeneity of lipoproteins, and method of *in vitro* oxidation. Studies have investigated oxLDL binding affinity to several ECM proteins, such as varying collagen types, laminin, fibronectin, and PDL. Jimi et al. described that copper-oxidized low-density lipoproteins (LDLs) had a higher binding affinity to individual matrix proteins compared to human native LDL (non-oxidized); this study also characterized that oxLDL binds more to type I (52%) and type III (48%) collagens when compared to type IV (35%) and V (13%) in two days (48). Greilberger et al. and Jimi et al. suggested that the negative charge of oxLDL allows it to bind to positively-charged regions of collagen (48, 49). Kalant et al. further indicated that LDL-collagen binding is considerably decreased when cultured with DMEM due to the presence of histidine in the medium (50). This could definitely explain why pre-treatment (48 h) of rat-tail COL-I prior to fabrication of hydrogels has higher binding affinity compared to direct integration into hydrogel matrix, which contains a 3X DMEM solution. Moreover, the oxLDLs used in this study were commercially available oxLDLs (ThermoFisher) isolated from human plasma and experimentally oxidized using a copper-mediated process. Although copper-oxidation is most widely used, Horl et al. alternatively explored a novel ozone-oxidation method (51). Further, oxidative modification of LDL can also be generated using different metal ions, reactive oxygen species (ROS), lipoxygenase, and myeloperoxidase (52). Future implementation of isolated lipoprotein could explore such methods for oxidation prior to incorporation in the model. As such, there are several considerations necessary for lipoprotein integration, binding affinity, and oxidation methods for *in vitro* oxLDL studies.

Within the CAVD-on-a-chip models, this study identified the formation of hydroxyapatite mineralization. As previously described, hydroxyapatites are common mineralization found in the pathological deposition of minerals and organic compounds of diseases and have a Ca/P of 1.67 (19, 35, 53, 54). Examination of *ex vivo* human calcified aortic valve tissue revealed that calcifications are composed of a variety of calcium phosphates: monocalcium phosphates (Ca/P = 0.5), dicalcium phosphates (Ca/P = 1), and octacalcium phosphates (Ca/P = 1.33), leading to hydroxyapatite formation (33, 55, 56). Mendoza et al. explored the use of the CAVD-on-a-chip platform to drive calcification *in vitro*, where hydroxyapatite mineralization was not found regardless of culture time. However, here, the introduction of oxLDLs was capable of leading to hydroxyapatite creation in just two days without the need for inflammatory cytokines (ex. TGFβ or TNFα), and/or osteogenic medium (3, 6, 17, 29, 34, 57). Still, a combination approach using chondroitin sulfate, as studied previously, and oxLDL, as studied here, could be further investigated (24, 26). Additionally, this study analyzed culture changes in only 2 days; future work can look to extend culture time and further drive disease progression.

Our previous study indicated that 20 dyne/cm^2^ shear could induce high mineralization content (19); therefore, further analysis was performed to compare static to shear. Aortic valve endothelial cell layers are responsible for providing mechanical strength, elasticity, and structural integrity to withstand hemodynamic forces (39, 58). Models of arterial LDL accumulation demonstrated that endothelial permeability to LDL uptake is proportional to the LDL surface concentration and magnitude of shear stress, suggesting that shear stress-induced biological changes can affect LDL accumulation *in vitro* (59, 60). Specifically, shear related-endothelial to mesenchymal transformation (EndMT) has been seen in oxLDL-induced disease models. Kim et al. found that human aortic endothelial cells and atherosclerotic-prone apolipoprotein E-deficient mice tissues underwent radiation-induced induced EndMT that was then further accelerated by the deposition of oxLDL (61). Additionally, Yang et al. demonstrated that conditioned medium with cytokines released from oxLDL-treated M1 macrophages drove EndMT in human aortic atherosclerotic plaques and increased endothelial permeability (62). Although not studied here, previous *in vitro* (19, 24, 26) and computational (27) studies demonstrated that EndMT results in activated myofibroblastic phenotypes that remodel the ECM, causing fibrosis and calcification to occur closer to the endothelial cell layer. This model demonstrates early detection of shear related-EndMT as a potential mechanism driving endothelial permeability and matrix remodeling.

Furthermore, we also demonstrated evidence of endothelial dysfunction in cultures exposed to oxLDL treatment and in the presence of shear stress (20 dyne/cm^2^). Both qualitatively and quantitatively in SEM micrographs, we found evidence of endothelial injury characterized by fenestrations in their cell membranes. In a liver cell study with oxLDL treatment, Zhang et al. characterized how oxLDL similarly induced endothelial membrane injury through ROS formation and NFκB activation (36). Mundi et al. published a comprehensive review on the interaction between LDL and the endothelium, and driving cardiovascular disease (37). *In vivo*, lipoprotein trans-endothelial passage is identified by the glycocalyx, a dense matrix layer of glycoproteins, proteoglycans, and GAGs on the endothelial surface, and LDL then crosses the endothelium through vesicular transcytosis (37). *In vivo* studies in rats indicated that LDL also crosses the endothelium through “leaky junctions,” an inter-junctional space, associated with dying and dividing cells, and contributing to aortic endothelial permeability found in atherosclerosis (37, 63). Although most studies related to endothelial dysfunction focus on atherosclerosis, few studies look to explore endothelial stability, integrity, and function in CAVD (2, 39, 64). Poggianti et al. carried out a clinical systematic study indicating a strong correlation between aortic sclerosis and systemic endothelial dysfunction via endothelium-dependent, flow-mediated dilation of the brachial artery (65). The analysis of explanted human calcified aortic valves performed by Matsumoto et al. revealed a pathological link for the destruction of VECs with decreased levels of endothelial progenitor cells and an increase in senescence-associated β-galactosidase activity (64). Similarly, immunohistology or immunocytochemistry could be used to study changes in a disrupted endothelial layer (64). Tompkins et al. demonstrated that LDL and albumin molecular transport in aortic valves (*in vivo* rabbits and monkeys) is mediated by trans-endothelial permeability (38). Yang et al. tested endothelial dysfunction by culturing an endothelial monolayer with fluorescent dextran and analyzed permeability using fluorescent plate reading (62). Likewise, future work could benefit from further characterization of the PAVEC monolayer permeability as related to oxLDL treatment and spatial deposition of calcification.

Additionally, increased oxidative stress has been identified as a cardiovascular risk factor affecting endothelial permeability. Oxidative stress, caused by the imbalance of ROS production and antioxidants *in vivo*, has led to the initiation of atherosclerotic plaque formation (52). As described in the results, in both static and dynamic models, an increased concentration of oxLDLs present in the culture increased the endothelial cell pore frequency and size. Similarly, studies have reported the expression of osteogenic differentiation markers when VICs were treated with lipoproteins causing ROS-mediated calcification and demonstrating that ROS plays a vital role in the initiation and propagation of CAVD (66). Valente et al. concluded that oxLDL and LPC further enhanced the generation of superoxides in endothelial cells (43). Additionally, Dandapat et al. found that oxLDL integration increased the expression of NADPH oxidase and intracellular ROS generation in human coronary artery endothelial cells (67). Branchetti et al. artificially exposed human VICs to hydrogen peroxide, reporting that ROS resulted in the expression of RUNX2, an osteogenic signaling molecule, and DNA damage (68). Similarly, Salemizadehparizi et al. reported that co-cultured VICs and VECs exposed to ROS increased calcium concentration suggesting VEC-VIC crosstalk influences nodule maturation (69). Our study establishes a preliminary connection between endothelial presence and permeability, and oxidative stress *in vitro*. Additional studies are required to explore the interaction between lipoprotein deposition and oxidation, oxidative stress, and early CAVD.

This microfluidic platform is capable of withstanding high shear (20 dyne/cm^2^) and integrating oxLDL into the COL-I matrix. Building upon previously published work, this model found that oxLDL accumulation drives excess deposition of calcium and phosphate and thereby generating hydroxyapatite formation. The key takeaways of this study are: (1) oxLDL integration into a 3D microfluidic CAVD modeling platform, (2) generation of human-like calcification varying in calcium phosphate mineralization, including hydroxyapatites in 2 days under shear conditions, (3) porcine cells were able to uptake oxLDL *in vitro*, (4) the model showed evidence of dynamic endothelial dysfunction. Given that CAVD has no targeted therapeutic intervention, continued evolution of this model can lead to significant contributions in preclinical drug development.

## Data Availability

The raw data supporting the conclusions of this article will be made available by the authors, without undue reservation.
