# Supplementary material for: Oxidative low-density lipoprotein and shear induced calcification within a calcific aortic valve disease-on-a-chip platform
Source: Front Cardiovasc Med. 2025 Oct 23;12:1655341. doi: 10.3389/fcvm.2025.1655341 (PMC12589058; doi:10.3389/fcvm.2025.1655341)
Supplement: Supplementary file 1 [file Datasheet1.docx]

Supplementary Material

# Supplementary Figures and Tables

## Supplementary Figures


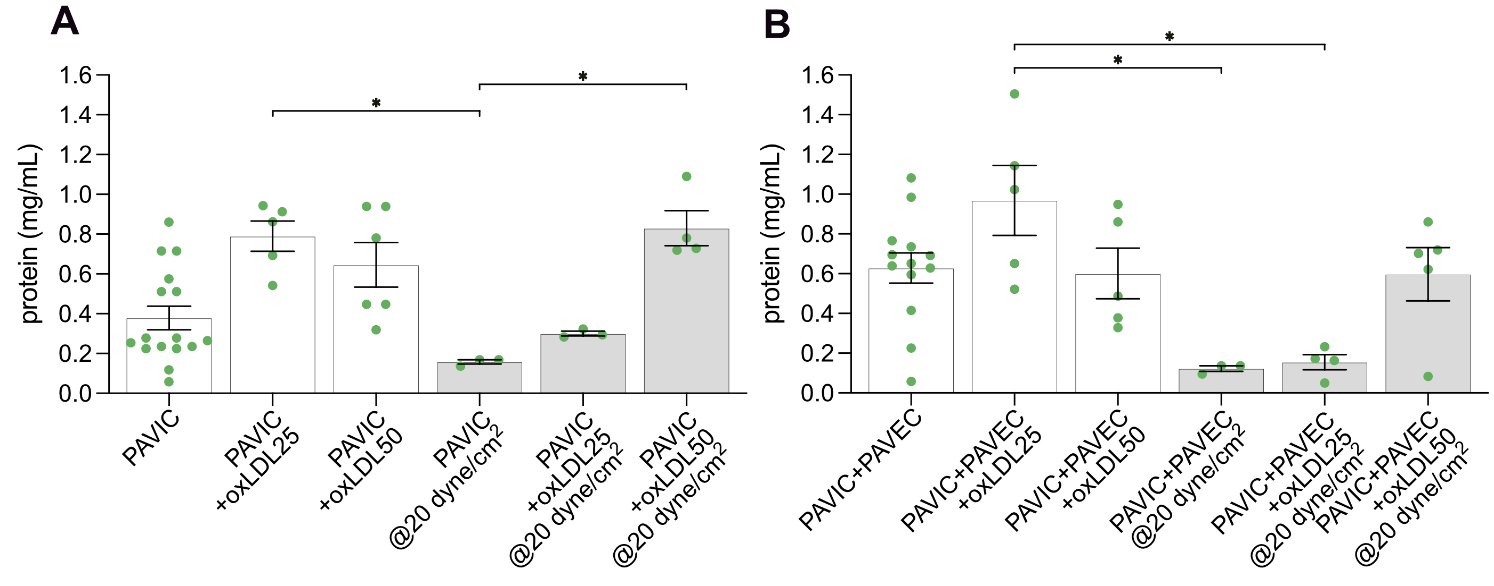


Supplementary Figure 1. Protein detection via Bradford assay after 2 days in culture: Static and dynamic collagen hydrogels with (A). PAVIC and (B). PAVIC/PAVEC co-culture. Data shown as mean ± SEM, where static (n≥5) and dynamic (n=3), and statistical significance shown according to Kruskal-Wallis with Dunn’s Multiple Comparisons *post-hoc* test, *p<0.05. (*PAVIC= Porcine aortic valve interstitial cells, PAVEC= Porcine aortic valve endothelial cells, oxLDL25= 25 µg/mL oxidative low-density lipoproteins, oxLDL50= 50 µg/mL oxidative low-density lipoproteins*).


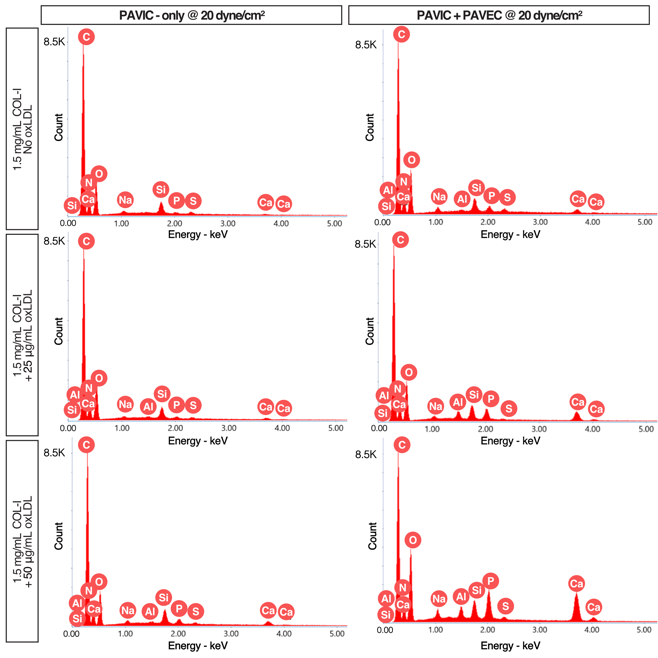


**Supplementary Figure** 2**.** **Microfluidic EDX spectra after 2 days in culture with PAVIC-only and when co-cultured with PAVEC, with increasing oxLDL concentration.** (*PAVIC= Porcine aortic valve interstitial cells, PAVEC= Porcine aortic valve endothelial cells, COL-I = collagen-I, oxLDL= oxidative low-density lipoproteins*).


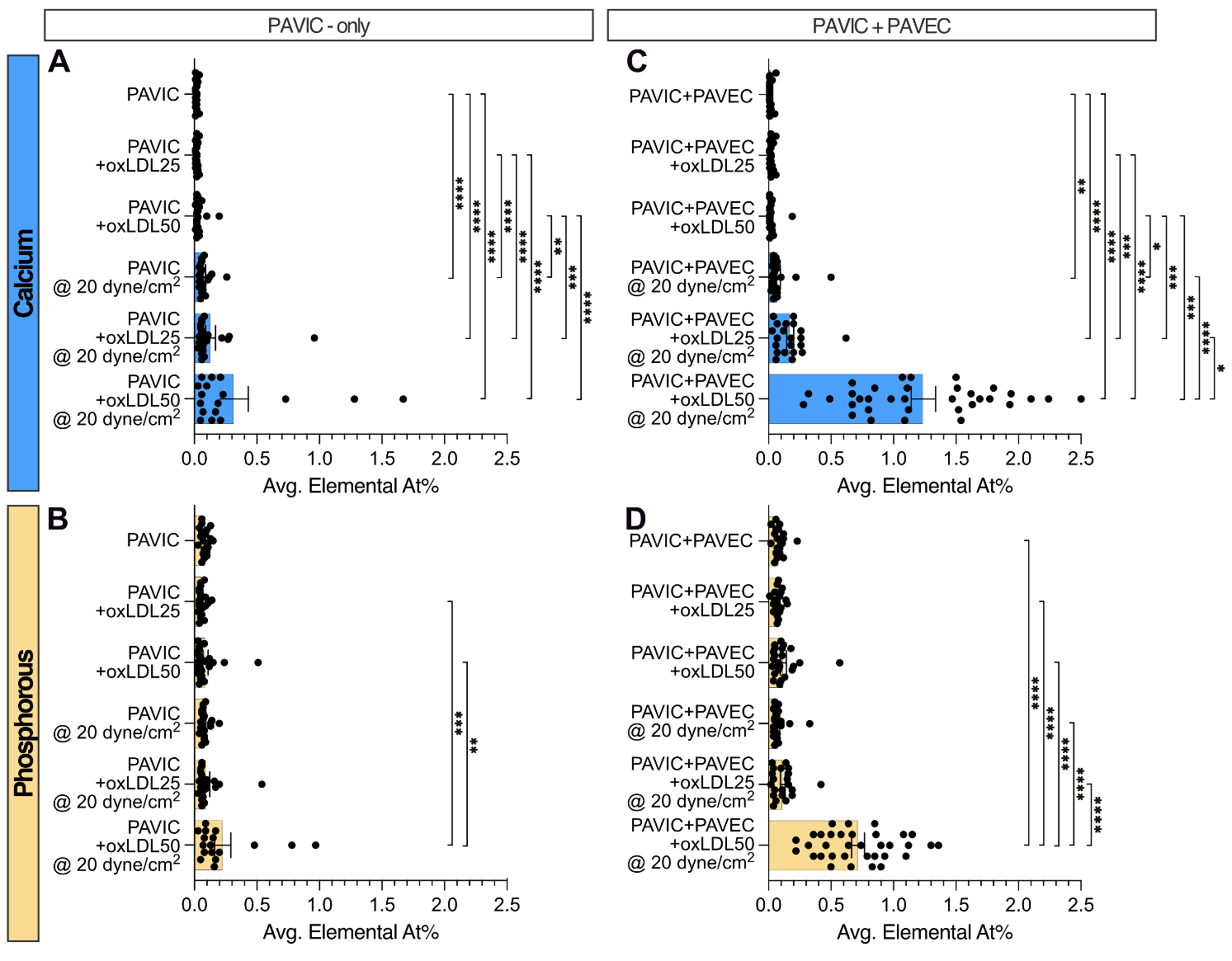


Supplementary Figure 3. Static and microfluidic EDX analyzes after 2days in culture, PAVIC-only and when co-cultured with PAVEC. PAVIC-only (A). calcium, (B). phosphorous, PAVIC+PAVEC co-cultures: (C). calcium, (D). phosphorous. Mean±SEM, n≥18 measurements. Statistical significance shown according to Kruskal-Wallis with Dunn’s Multiple Comparisons *post-hoc* test, *p<0.05. (*PAVIC= Porcine aortic valve interstitial cells, PAVEC= Porcine aortic valve endothelial cells, oxLDL25= 25 µg/mL oxidative low-density lipoproteins, oxLDL50= 50 µg/mL oxidative low-density lipoproteins*).


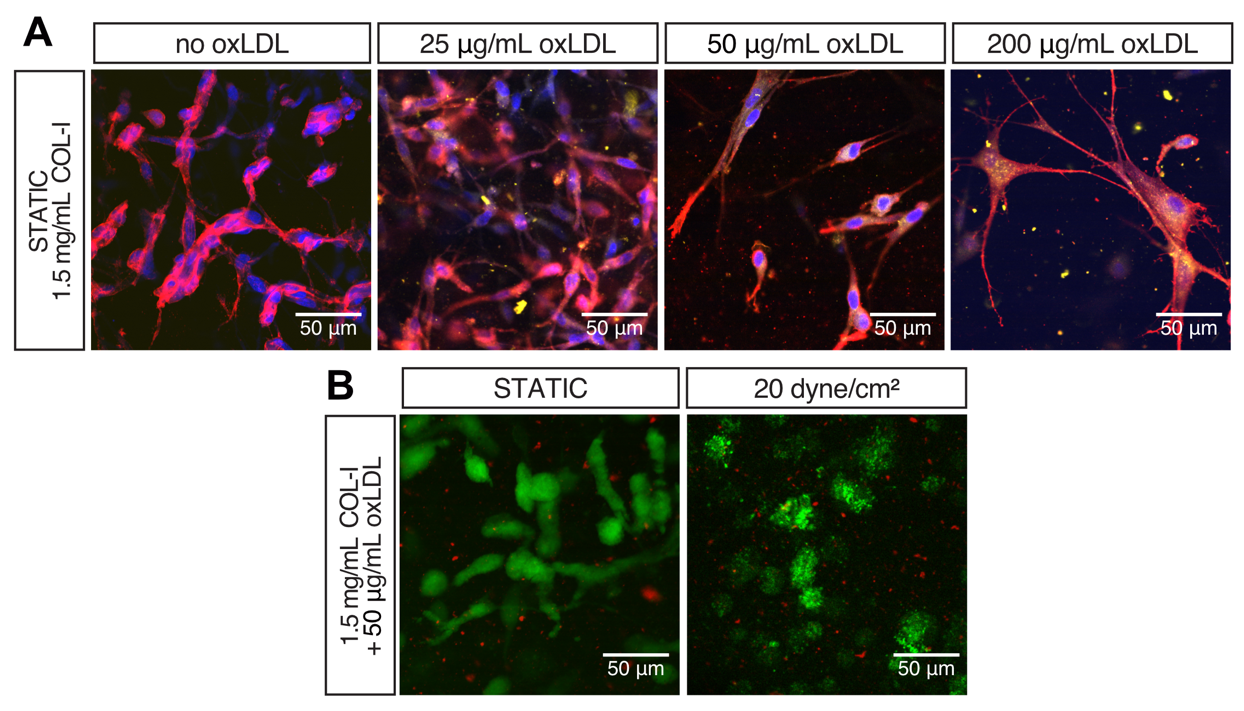


Supplementary Figure 4. Preliminary static PAVIC+PAVEC co-cultures with varying oxLDL concentrations after two days, (A). co-cultures with 25 µg/mL oxLDL, 50 µg/mL oxLDL, and 200 µg/mL oxLDL, Key: Plasma membrane (CellMask = Red), DNA (Hoechst = Blue), oxLDL (Dil-oxLDL = Yellow). (B). Live imaging of co-cultures with 50 µg/mL oxLDL, Key: Live (Calcein AM = green) and oxLDL (Dil-oxLDL = Red) (scale = 50 µm) (*COL-I = collagen-I, PAVIC= Porcine aortic valve interstitial cells, PAVEC= Porcine aortic valve endothelial cells, oxLDL= oxidative low-density lipoproteins*).
